# Is diabetic ketoacidosis a good predictor of 5-year metabolic control in children with newly diagnosed type 1 diabetes?

**DOI:** 10.1186/s12902-021-00882-8

**Published:** 2021-11-02

**Authors:** Kowalczyk Emilia, Stypułkowska Aneta, Majewska Barbara, Jarzębowska Małgorzata, Hoffmann Aleksandra, Buła Paulina, Szypowska Agnieszka

**Affiliations:** 1 Department of Pediatric Diabetology and Pediatrics, Pediatric Teaching Clinical Hospital of the Medical University of Warsaw, Żwirki i Wigury 63A, 02-091 Warsaw, Poland; 2 Students’ Scientific Association, Medical University of Warsaw, Warsaw, Poland; 3 Department of Pediatrics, Medical University of Warsaw, Warsaw, Poland

**Keywords:** In-patient diabetes, Children and adolescents, Other complications, Devices, Self-management

## Abstract

**Background:**

The study aimed to evaluate whether the presence of DKA at diabetes diagnosis was associated with poorer metabolic control during a 5-year follow-up.

**Methods:**

The study included children treated due to newly diagnosed T1D complicated with DKA between 2010 and 2014 with a complete 5-year follow-up. In every case we performed individual matching for age, gender and BMI with a person without DKA (nDKA) on recognition. We collected data regarding treatment modality, HbA_1C_, total daily insulin dose, basal insulin and BMI-SDS.

**Results:**

85 children at the median age of 7.93 years had DKA at diabetes diagnosis. The median pH was 7.19.Continuous subcutaneous insulin infusion (CSII) was used in 87% of participants in each group. No differences in HbA_1C_ level (7,3%vs7,2%;*p* = .413) were noted after 5 years of disease duration. The severity of ketoacidosis exerted no significant effect on HbA_1C_. The method of insulin delivery at baseline was significantly associated with HbA_1C_ levels after 5 years of observation, β_CSII_ = − 1.46,95%CI[− 2.01 to − 0.92],*p* < .001.

**Conclusions:**

The presence of DKA at diabetes diagnosis is not associated with deteriorated long-term metabolic control in children using modern technologies. The early implementation of CSII into diabetes treatment may change the effect of DKA and lead to a long-term HbA_1C_ improvement.

## Background

Type 1 diabetes (T1D) is one of the most common metabolic diseases in the pediatric population worldwide. The global increase in the incidence of type 1 diabetes among children still remains at the level of 3 to 5% annually [1, 2]. The alarming epidemiological situation may also be observed in Poland with the tendency toward younger age groups [3]. In children aged 0–14 the disease frequency boosted approximately six-fold from 4.2 per 100,000 persons/year in the early 1970s to 24.3 per 100,000 persons/year in 2013 with regular, sinusoidal fluctuations and a slight levelling-off over the past few years [3–5].

.The International Society for Pediatric and Adolescent Diabetes (ISPAD) stated that diabetic ketoacidosis (DKA) was the most common, but potentially preventable life-threatening complication related to newly diagnosed T1D [6]. The incidence of this problem is still unacceptably high, especially in the youngest group (below 5 years) and has not changed over the last 20 years despite a sharp increase in T1D diagnoses and improvement in medical care [1, 2, 7]. Overall, more than one-third of newly diagnosed children are affected by DKA [2, 7–9]. A large global variation is observed in DKA prevalence, with the values varying from 14.7% (Denmark) to 79,8% (Saudi Arabia) depending on a geographic location, the socioeconomic factors of the country and the incidence of diabetes (the awareness is poorer in countries with a lower incidence) [2]. The socioeconomic inequalities play a crucial and important role in the DKA rate at diabetes diagnosis [2, 7–9]. The frequency of DKA in Poland was estimated to be between 22 to 36% [1, 10–12] and the incidence of coma due to DKA was around 4.7% of children [12]. DKA is the most common cause of diabetes-related death and has an associated mortality rate from 0.15 to 0.35% in developed countries and from 3.4 to 24% in developing countries [7]. Cerebral edema is the leading cause of DKA-related death. It occurs in 0.3–1% people at disease onset [3]. Some authors suggested that when clinically cerebral edema was not observed mild brain injuries were present in at least 50% of children during DKA treatment [13]. Negative neurological complications were associated with persistent alterations in attention and memory 6 months following a DKA episode. Moreover, DKA severity and younger age were the greatest risk factors for changes in the cerebral structure [14]. Aye et al. reported that a single episode of moderate or severe DKA in young children at diabetes diagnosis contributed to lowering the cognitive function and altering brain growth [2, 15]. DKA was also correlated with a longer hospitalization and higher baseline insulin requirements [16]. Moreover, ketoacidosis at diabetes onset was associated with a poor residual β-cell function and, thereby, a shorter remission period [1, 3]. Data concerning long-term glycemic control depending on DKA presence on diabetes diagnosis are contradictory. The majority of authors supported the concept of metabolic tracking and reported a negative influence on metabolic control if diabetes was accompanied by DKA at onset [2, 3]. Numerous differences are observed worldwide in terms of the access to medical services, education level, approach to new insulin and diabetes technologies which could influence the observed data inhomogeneity. No reports concerning this field are available in Poland.

The major objective of our study was to assess whether long-term glycemic control in children and adolescents with T1D who had DKA at diabetes onset was poorer than in people with T1D without DKA during a 5-year observation period.

## Methods

The study population included people treated in the Department of Pediatric Diabetology and Pediatrics, Pediatric Teaching Clinical Hospital of the Medical University of Warsaw due to newly diagnosed type 1 diabetes complicated by DKA between 2010 and 2014. The inclusion criteria were: newly recognized diabetes type 1 with DKA at the onset of the disease and a complete follow-up for 5 consecutive years. No restrictions on participation were imposed as regards the type of insulin therapy. Participants treated both with continuous subcutaneous insulin infusion (CSII) and multiple daily injections (MDI) were recruited. Treatment with insulin pumps providing continuous subcutaneous insulin infusion has been available to children with diabetes mellitus in Poland since 2008. The service is covered by the National Health Fund (Narodowy Fundusz Zdrowia, NFZ) from the catalogue of public-funded services under separately commissioned healthcare services. The patients had the access to two types of insulin pump brands: Medtronic (MiniMed) and Roche - Accu Check. A tender for insulin pumps is conducted annually by the hospital and the access to the devices depends on its results. Most participants had CSII implemented at diabetes diagnosis. Personal continuous glucose monitoring devices have been widely available in Poland since 2018 (70% of the price of the devices was paid by the national health insurance system covered by public funds). Closed loop pumps were not available for any study participants.

We excluded participants diagnosed and initially treated in another Pediatric Department who were finally transferred to our Department and those with an incomplete follow-up. Subsequently, individual 1:1 matching was performed: every participant with DKA was matched with a newly diagnosed type 1 diabetes person without DKA. The participants were matched for age, gender and Body Mass Index (BMI) to minimize the influence of residual confounding. Moreover, no differences were revealed in health insurance status. Polish citizens have equal and unpaid access to healthcare services provided by the national health insurance system covered by public funds and managed by NFZ. DKA was defined as pH below 7.3 with concomitant hyperglycemia exceeding 200 mg/dL (11 mmol/L) and ketonemia or ketonuria [6]. The autoimmune origin of diabetes was confirmed with typical autoantibody testing and C-peptide level measurement. The onset was defined as the first 2 weeks after T1D manifestation. The obligatory part of the first hospitalization was a 2-week intensive educational course with a diabetic educator.

Data were collected at diagnosis and prospectively, during routine clinical visits for 5 years following the diagnosis. During the observation period the participants were under the constant care of the Outpatient Clinic (every 3 months) and had permanent access to medical assistance. Data concerning HbA_1C_, insulin requirements (the total daily dose of insulin (TDD), basal rate) and Body Mass Index standard deviation score (BMI-SDS) were collected during every clinical visit. BMI-SDS was calculated using the World Health Organization (WHO) child growth standards. The once-yearly assessment of metabolic control was selected and analyzed in every participant. We took into consideration the visit according to the date of diagnosis, and the dates which were the closest to the date of diagnosis + 1 year, + 2 years (etc.).

The follow-up period was set for 5 years and only data from those who completed the follow-up were investigated. The comparison of HbA_1C_ between DKA and nDKA groups after 5 years of treatment constituted the primary outcome. The secondary outcomes included the comparisons of the following parameters between groups: TDD, basal rate and BMI-SDS. The analysis was also performed in subgroups with different degrees of acidosis defined according to the ISPAD [6] criteria: mild, moderate, severe (pH < 7.3; < 7.2; < 7.1; respectively). Good metabolic control was defined as HbA_1C_ ≤ 6.5% according to the ISPAD target of treatment [17].

### Laboratory work-up

At diabetes diagnosis standard laboratory methods were used to measure glucose and blood gases in the clinical laboratory of the Pediatric Teaching Clinical Hospital of the Medical University of Warsaw. HbA_1C_ levels were measured at baseline and at each visit in the Outpatient Clinic with high-performance liquid chromatography (D-10 Hemoglobin Testing System, Bio-Rad Laboratories, USA) with a nondiabetic range of 4.1–6.4% (21–46 mmol/mol).

The Children’s Memorial Health Institute Laboratory performed autoantibody testing to glutamic acid decarboxylase (GADA), islet cell antibodies (ICA, against cytoplasmic proteins in the beta cell) and insulinoma-associated-2 autoantibodies (IA-2A). Fasting C-peptide concentrations were analyzed using radioimmunoassay, with an assay detection limit of 0.1 nmol/L.

### Statistical analysis

GraphPad Prism Software Version 8.4.2 was used for all analyses. Nominal variables were presented as n (% of group), continuous variables as medians (Q1;Q3) due to the lack of normal distribution. Data normality was verified based on the Shapiro-Wilk test. Spearman’s rank correlation tests were conducted to examine the correlations between baseline pH and HbA_1C_ Univariate linear regression analysis was conducted to investigate the influence of CSII/MDI on the 5th year HbA_1C_. Model β coefficient with 95% confidence intervals (CI) was estimated.

Group comparison for nominal variables was conducted with the Fisher’s exact test or the χ2 test, as appropriate. The Wilcoxon signed rank test was used for the paired comparisons of clustered data. Continuous variables were compared with the Kruskal-Wallis and the Mann–Whitney *U* test. All tests were two-tailed, and the differences were considered significant at the level of *p* value of ≤0.05.

## Results

A total of 127 potentially eligible participants were recruited. 42 participants were excluded due to missing data, an incomplete follow-up period or transfer from pediatric to adult medical care. As a result, 85 individuals met the study inclusion criteria and were analyzed. DKA participants were matched 1:1 to 85 participants without DKA at diagnosis. The baseline characteristics of the study groups are presented in Table 1. The year of diagnosis with number of cases per year in each group are shown in Table 2.

**Table 1:** Characteristic of study groups

	Participants without DKA (***n*** = 85)	Participants with DKA (***n*** = 85)	***p***-value
**Gender (F/M), n**	37/48	37/48	
**Age, years**	7.63 (5.81;10.52)	7.93 (4.30;10.42)	.430
**pH on recognition**	7.39 (7.35;7.42)	7.19 (7.10;7.27)	< 0.001
**HbA**_**1C**_ **on diagnosis of diabetes, %**	11.30 (10.05;12.75)	12.50 (11.2;14.00)	< .001
**HbA**_**1C**_ **on diagnosis of diabetes, mmol/mol**	100.00 (86.00;116.00)	113.00 (99.00;130.00)	< .001
**BMI-SDS**	−0.26 (− 1.32;0.59)	−0.55 (− 1.75;0.51)	.156
**CSII, n%**	74 (87)	74 (87)	
**TDD, u/kg**	0.40 (0.25;0.59)	0.51 (0.34;0.69)	.005
**Base, u/kg**	0.05 (0.02;0.09)	0.09 (0.07;0.13)	< .001

*Abbreviations*: *DKA* diabetic ketoacidosis group; n-DK, *F* female, *M* male, *HbA*_*1C*_ glycated hemoglobin, *n* number of participants, *TDD* Total Daily Dose of Insulin, *Basal Rate* %TDD covered by basal insulin, *u/kg* Unit per kilogram, *BMI-SDS* Body Mass Index Standard Deviation Score

**Table 2:** The dates of diagnosis and the number of cases per year in each group

YEAR	2010	2011	2012	2013	2014
DKA group [n]	12	13	24	26	10
nDKA group [n]	13	17	21	22	12

*Abbreviations: DKA* diabetic ketoacidosis group, *nDKA* non-diabetic ketoacidosis group, *n* number of subjects

85 children at the median age of 7.93 years had DKA at diabetes diagnosis with the median pH of 7.19. Severe DKA occurred in 20 participants (24%), moderate in 23 (27%) and mild in around half of DKA group (49%). HbA_1C_ at onset of diabetes was statistically higher in DKA children (*p* = < .001) with the maximum values reported in those with severe DKA (122 mmol/mol; 13.25%). Insulin pump therapy was implemented in the majority of participants during the first hospitalization, within 2 weeks after the stabilization of the general condition. The same insulin therapy paradigm was introduced, as 87% (*n* = 74) of the participants in each group used CSII. During the follow-up period 1 person from nDKA group changed insulin therapy from CSII to MDI after 1 year. Children in DKA group had higher TDD (*p* = .005) and basal insulin (*p* < .001) compared to nDKA participants. A moderate positive correlation was revealed between initial HbA_1C_ and basal insulin requirement (*r* = 0.33; *p* < .001).

**Fig. 1:**
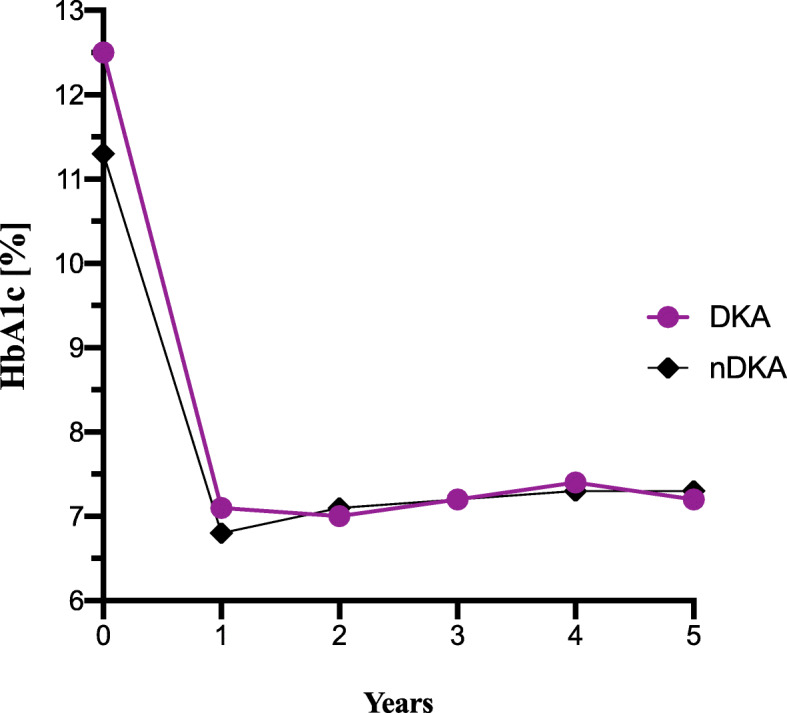
HbA_1C_ change during observation period in both groups. Abbreviations: DKA, diabetic ketoacidosis group; nDKA, non- diabetic ketoacidosis group; HbA_1C_, glycated hemoglobin

A rapid and sharp decline in HbA_1C_ value was observed after the first year following the diagnosis of diabetes. Then, it remained stable at a similar level for the next 4 years in both groups (Fig. 1). After 5 years of the disease HbA_1C_ level was similar regardless of the group (*p* = .413). The 5th year HbA1c was not correlated with baseline HbA_1C_ (*r* = 0.055; *p* = .478) and baseline pH (*r* = 0.066; *p* = .396). However, a correlation was noted between the 5th year HbA_1C_ and age at diagnosis (*r* = 0.204; 95%CI 0.06–0.34; *p* = .004). A univariate linear regression model showed that the method of insulin delivery at baseline (CSII or MDI) was significantly associated with the 5th year HbA_1C_, β_CSII_ = − 1.46, 95%CI [− 2.01 to − 0.92], *p* < .001.

**Fig. 2:**
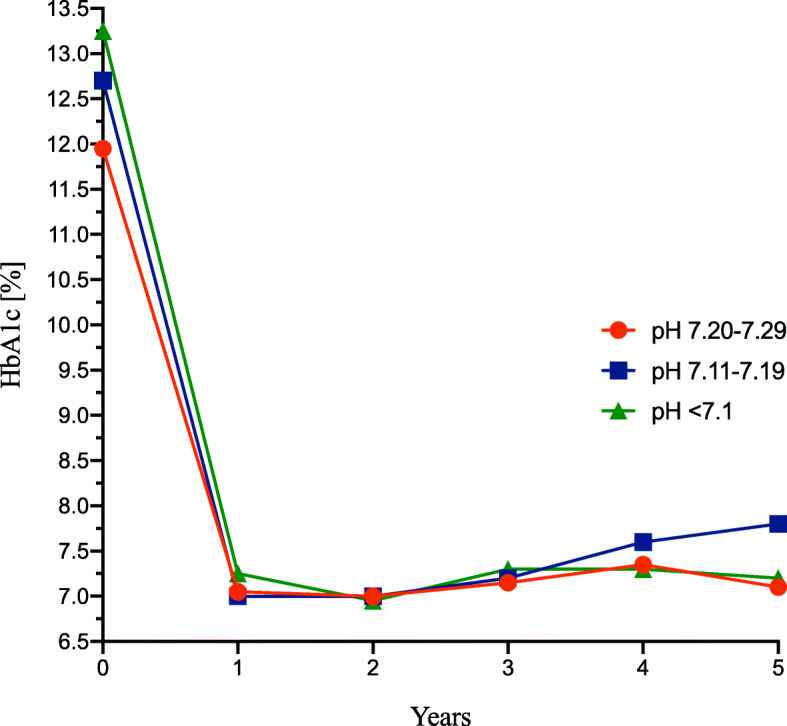
HbA_1C_ change during observation period in DKA group. The presentation in subgroups with different degrees of acidosis (pH). Abbreviations: HbA_1C_, glycated hemoglobin; DKA, diabetic ketoacidosis group

The 5th year HbA_1C_ was the highest among children with moderate acidosis (62 mmol/mol; 7.80%) in comparison with mild (54 mmol/mol; 7.10%) and severe (55 mmol/mol; 7.20%), but without statistical significance (*p* = .085) (Fig. 2).

The number of participants who achieved good metabolic control (HbA_1C_ ≤ 6.5%) was 10% greater in nDKA compared to DKA group after the first year of the disease (38% vs 28%, respectively; *p* = .253). After 5 years of follow-up it remained at a similar level (18% vs 22%, respectively; *p* = .566). A similar analysis was performed in terms of poor metabolic control (HbA_1C_ ≥ 8%). A similar rate of participants in both groups achieved the elevated HbA_1C_ level after the first year of the disease (nDKA 16.5% vs DKA 12.9%, respectively; *p* = .666) and after 5 years of follow-up (nDKA 24.7% vs 30.6%, respectively; *p* = .493).

Total daily insulin requirements were similar in both groups after the follow-up period (*p* = .629). Basal insulin requirements at diagnosis and after the first year of the disease were greater in those who had acidosis at the onset of diabetes. No changes concerning insulin requirements were observed in relation to DKA severity. An interesting trend was observed in relation to BMI z-score. Children who had acidosis at diagnosis featured lower body weight at the beginning and rapid growth afterwards. Their body weight increased to a higher extent than in nDKA group individuals over the next 4 years. However, the observed differences were not statistically significant. Furthermore, no significant differences were noted in terms of metabolic control (HbA_1C_, insulin requirements, body weight) 5 years after T1D diagnosis between age groups and genders in DKA and nDKA groups. The main outcomes were presented in Table 3.

**Table 3:** Comparison between groups during follow-up

	Participants without DKA (***n*** = 85)	Participants with DKA (***n*** = 85)	***p***-value
**1 year after diagnosis of diabetes**			
HbA_1C_, %	6.80 (6.20;7.40)	7.10 (6.50;7.70)	.233
HbA_1C_, mmol/mol	51.00 (44.00;57.00)	54.00 (4800;6100)	
BMI-SDS	0.29 (−0.54;0.91)	0.45 (0.01;1.26)	.131
TDD, u/kg	0.58 (0.41;0.75)	0.63 (0.50;0.75)	.220
Base, u/kg	0.11 (0.06;0.19)	0.16 (0.11;0.22)	**.003**
**2 years after diagnosis of diabetes**			
HbA_1C,_ %	7.10 (6.60;7.85)	7.00 (6.60;7.50)	.676
HbA_1C_, mmol/mol	54.00 (49.00;62.00)	53.00 (49.00;58.00)	
BMI-SDS	0.40 (−0.34;1.10)	0.45 (0.01;1.26)	.155
TDD, u/kg	0.79 (0.62;0.91)	0.74 (0.61;0.86)	.436
Base, u/kg	0.19 (0.12;0.26)	0.22 (0.15;0.28)	.194
**3 years after diagnosis of diabetes**			
HbA_1C,_ %	7.20 (6.70;7.80)	7.20 (6.60;7.70)	.522
HbA_1C_, mmol/mol	55.00 (50.00;62.00)	55.00 (49.00;61.00)	
BMI-SDS	0.43 (−0.42;1.05)	0.53 (−0.24;1.00)	.604
TDD, u/kg	0.80 (0.64;0.93)	0.78 (0.69;0.93)	.831
Base, u/kg	0.23 (0.16;0.32)	0.27 (0.18;0.31)	.184
**4 years after diagnosis of diabetes**			
HbA_1C,_ %	7.30 (6.85;8.00)	7.40 (6.80;8.25)	.983
HbA_1C_, mmol/mol	56.00 (51.00;64.00)	57.00 (51.00;67.00)	
BMI-SDS	0.40 (−0.50;1.10)	0.65 (0.01;1.15)	.100
TDD (u/kg)	0.81 (0.67;0.97)	0.80 (0.70;0.97)	.936
Base (u/kg)	0.25 (0.20;0.34)	0.27 (0.22;0.33)	**< .001**
**5 years after diagnosis of diabetes**			
HbA_1C,_ %	7.30 (6.75;8.00)	7.20 (6.60;8.10)	.413
HbA_1C_, mmol/mol	56.00 (50.00;64.00)	55.00 (49.00;65.00)	
BMI-SDS	0.40 (−0.29;1.08)	0.45 (−0.10;1.17)	.546
TDD (u/kg)	0.84 (0.74;0.96)	0.80 (0.72;0.97)	.629
Base (u/kg)	0.28 (0.21;0.36)	0.29 (0.23;0.34)	.579

*Abbreviations*: *DKA* diabetic ketoacidosis group; n-DKA, *HbA*_*1C*_ glycated hemoglobin, *n* number of participants, *TDD* Total Daily Dose of Insulin, *u/kg* Unit per kilogram, *BMI-SDS* Body Mass Index Standard Deviation Score

## Discussion

To the best of our knowledge, it is the first study in Poland investigating whether DKA at diabetes diagnosis is associated with long-term metabolic consequences. Our research was designed in a unique manner with individual matching to eliminate confounding and focus only on the impact of DKA on metabolic control. The results showed that DKA at diabetes onset was not associated with poorer HbA_1C_, greater insulin requirements and higher body weight for 5 years of the disease. HbA_1C_ was similar in DKA and nDKA groups throughout the 5-year follow-up.

The findings of our research are consistent with a study conducted by Khanolkar et al. [18] The authors analyzed associations between DKA presence at diagnosis and glycemic control during the first year post-diagnosis in 341 children and found that DKA was not correlated with deteriorated metabolic control [18]. Piccini et al. conducted a study based on the SWEET registry to investigate if the presence of DKA or HbA_1C_ level at diabetes onset was a better predictor of long-term metabolic control and concluded that the 3rd year HbA_1C_ was more closely related to baseline HbA_1C_ than to DKA presence at onset [12]. The authors concluded that children who experienced DKA at diagnosis might achieve good metabolic control, but those with high primary HbA_1C_ continued to have poor metabolic control [12]. The present study showed no correlation between the 5th year HbA_1C_ and HbA_1C_ or pH at diabetes diagnosis. Data concerning the influence of DKA on long-term metabolic control are conflicting, but most of them relate to poor long-term prognosis if acidosis was present at diagnosis. The unfavorable impact of initial DKA was reported in a study by Shalitin et al. conducted after the first year post-diagnosis. The mean daily insulin dose (0.74 ± 0.26 vs 0.69 ± 0.27 units/kg/d, *p* = .049) and HbA_1C_ level (7.85 ± 1.13% vs 7.49 ± 0.94%, *p* = .01) were significantly higher in participants with DKA at diagnosis [19]. Our study showed that only basal insulin dose was significantly higher in DKA group after the first year of follow-up. Analogous negative effects of DKA were demonstrated in a cohort study based on data retrieved from DanDiabKids, a Danish national diabetes registry of children with newly diagnosed type 1 diabetes. It comprised 2964 children with a long-term follow-up (5.8 years on average) [20]. They found moderate and severe DKA to be associated with increased HbA_1C_ (0.24; 95% CI 0.11,0.36; *p* = .0003) and insulin dose-adjusted HbA_1C_ during the observation period [20]. Interestingly, they noted that CSII therapy in comparison to MDI changed the effect of DKA at onset and led to the long-term improvement in HbA_1C_. The CSII therapy was implemented in around 40% of participants in the described study group [20].

.A factor which could influence the study results is a low number of children using multiple daily injections in both groups. We would like to highlight the fact that we recruited a unique population, as around 90% of the participants (the same number in both groups) were treated with CSII. The therapy was implemented just after diabetes diagnosis, during the first month, which probably affected the results we achieved. We noted that the method of insulin delivery at baseline (CSII or MDI) was significantly associated with the 5th-year HbA_1C_.

In the population-based cohort study conducted in 446 diabetes centers the data from 9814 patients using pump therapy were collected and matched with the same number of patients using injection therapy. Pump therapy, compared to injection therapy, was associated with lower rates of severe hypoglycemia and diabetic ketoacidosis. Glycated hemoglobin levels and total daily insulin doses were lower with pump therapy than with injection therapy. There was no significant difference in body mass index between both treatment regimens [21]. A study conducted by Piechowiak et al. in our Pediatric Diabetology Department evaluated factors influencing diabetes control over 3 years following diabetes diagnosis [22]. The researchers reported that children who were started on CSII at T1D onset achieved long-term optimal glycemic control [22]. A study based on Diabetes-Patienten-Verlaufsdokumentation (DPV) registry provided evidence for improved clinical outcomes associated with the early initiation (within the first 6 months) versus later implementation of insulin pump therapy in children with type 1 diabetes [23]. Seemingly, the early implementation of CSII might mitigate the negative consequences and all the burden of DKA at diabetes onset. Nevertheless, because of a great disproportion between groups we should be cautious in drawing far-reaching conclusions.

Interestingly, Piccini et al. found a positive implication between initial DKA and further metabolic control. DKA presence at diabetes diagnosis might frighten families and stimulate them to adhere to the diabetes regimen more strictly. Such an exception was reported in Northern Europe (Poland was included): after the 3rd year of the disease HbA_1C_ was even better in children who experienced DKA with a coma compared to children with DKA without a coma and with no DKA [12]. The findings might reflect the low coma rate related to DKA, a more intensive insulin treatment and high social awareness of the disease in those countries. It could also support and explain our results.

Moreover, similarly to some authors, we noted that participants who presented with DKA had a tendency to higher BMI-SDS after an initial lower rate [12]. We established no statistically significant differences between groups concerning BMI-SDS. Weight ‘differences’ at baseline are not surprising and probably reflect differences in disease severity (and the degree of weight lost in the preceding months). In order to avoid confusion, we matched participants from DKA group based on their weight taken at discharge, when the clinical status was stable, without acidosis and dehydration. Previously conducted research revealed a difference after the 3rd year favoring patients with T1D without DKA on recognition [12].

Race, ethnicity, socioeconomic status, and family structure are complex, interrelated variables that influence diabetes control. We recruited a homogenous population of children according to the race – all participants were Caucasian. The limitation of our study is associated with the lack of socioeconomic factor analysis. Numerous study results unequivocally demonstrate that some factors negatively impact diabetes metabolic control in children. Low socioeconomic status, lower levels of maternal education and maternal death are associated with poor metabolic control [24]. Interesting results were found in Danish children where a universal access to health care is provided. Inequality in glycemic control was noted to be related to maternal educational level among children with type 1 diabetes, i.e. lower maternal educational level was associated with poorer glycemic control [25].

## STRENGHTS and limitations

- The strength of our study is related to the unique population consisting of children with T1D treated mostly using CSII in around 90% of each group. Insulin pump therapy was implemented at diabetes onset which was rare at that time. Moreover, the study population was precisely matched (1:1) to eliminate confounding factors and focus only on the impact of DKA on the metabolic control with long-term follow-up.
- The limitation of this study is associated with the lack of socioeconomic factors and a low number of children using multiple daily injections in both groups.

## Conclusions

- The presence of DKA at diabetes diagnosis was not associated with poor long-term metabolic control in T1D children using modern technologies after T1D onset. CSII therapy introduced at diabetes onset might prevent HbA_1C_ deterioration during the follow-up period in children with DKA at diabetes diagnosis. Further studies are needed to evaluate the benefits of the early implementation of CSII to the long-term metabolic control in children with T1D complicated by DKA on recognition.

## Data Availability

Data available upon request. Please contact Emilia Kowalczyk, emilia.kowalczyk@uckwum.pl.
